# Taste for Blood: Hemoglobin as a Nutrient Source for Pathogens

**DOI:** 10.1371/journal.ppat.1002535

**Published:** 2012-03-08

**Authors:** Gleb Pishchany, Eric P. Skaar

**Affiliations:** Department of Pathology, Microbiology, and Immunology, Vanderbilt University Medical School, Nashville, Tennessee, United States of America; Duke University Medical Center, United States of America

## Introduction

Hemoglobin, which gives blood its red color, is perhaps the most recognized and well studied protein in nature. It is also a critical molecule during infection, as many microbes rely on hemoglobin to grow within their hosts. Here, we review the importance of hemoglobin to vertebrate physiology and how humans attempt to conceal hemoglobin from invading pathogens. We also provide examples of the elaborate mechanisms employed by microbes to acquire hemoglobin during infection. Finally, we discuss how genetic variations within hemoglobin affect susceptibility to infectious diseases.

## Hemoglobin Metabolism within Humans

To understand how hemoglobin is utilized by invading pathogens, one must understand the structure, function, and metabolism of this molecule. Hemoglobin is a tetrameric globular protein consisting of two alpha and two beta chains [1]. The alpha and beta chains are encoded by different loci and are differentially regulated during human development. Each of the four chains of hemoglobin encloses an iron-containing heme co-factor responsible for oxygen binding [2]. The main function of hemoglobin is to capture atmospheric oxygen and deliver it to host tissues for respiration. Hemoglobin is synthesized in developing red blood cells, which lose their nuclei and cease protein synthesis upon maturation. In healthy adults, hemoglobin constitutes one-third of total erythrocyte mass and approximately 15% of the whole blood mass [2]. Mature erythrocytes circulate in the blood for approximately three months, whereupon they become senescent and are removed from the bloodstream by macrophages. Hemoglobin from senescent erythrocytes is digested to facilitate the recycling of heme-iron. In the case of erythrocyte lysis, liberated hemoglobin is captured by the plasma protein haptoglobin to prevent oxidative damage inflicted by hemoglobin. The haptoglobin-hemoglobin complex is recognized by macrophages and removed from the plasma. Any free heme that is released from hemoglobin extracellularly is rapidly bound by another plasma protein known as hemopexin. The above strategies for hemoglobin and heme removal limit the toxicity associated with these molecules, ensure iron homeostasis, and prevent microbial growth.

## Hemoglobin as a Source of Iron to Invading Pathogens

**Figure 1:**
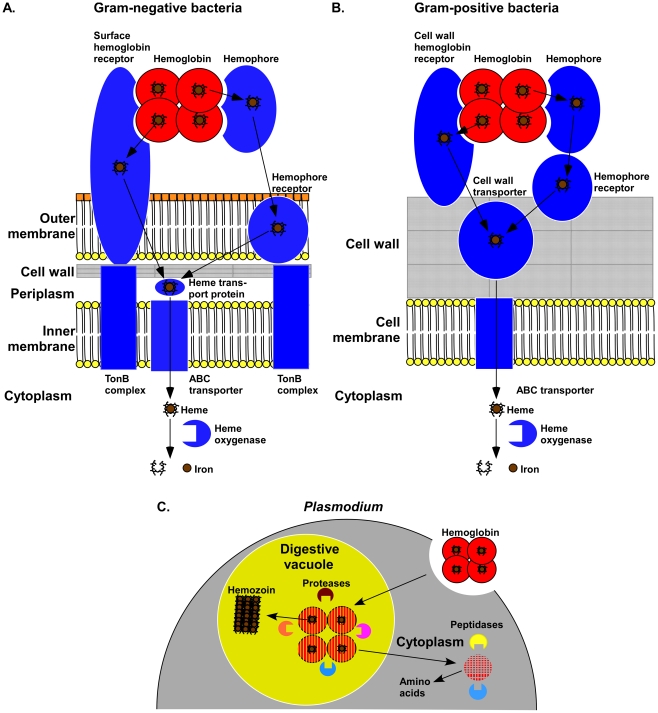
Mechanisms of hemoglobin utilization by pathogens. (A) Gram-negative bacteria bind hemoglobin by either secreted or surface receptors. Hemoglobin receptors extract heme and pass it to heme transport proteins within the cell surface. Outer membrane heme transport systems utilize the energy of the proton motive force generated within the inner membrane by the TonB complex. Once in the periplasm, heme is bound by a heme transport protein, which delivers heme to the inner membrane ABC transporter. ABC transporters pass heme into the cytoplasm, where it is degraded by heme oxygenases to supply the bacterium with iron. (B) Gram-positive bacteria express secreted and cell wall–anchored hemoglobin receptors that extract heme from hemoglobin. Hemoglobin receptors then transfer heme to the cell wall transporters that pass heme through the peptidoglycan layer in a relay process. Heme is then transported across the cell membrane by ABC transporters. In the cytoplasm, heme is degraded by heme oxygenases to release iron. (C) *Plasmodium* consumes hemoglobin by endocytosis of pockets of red blood cell cytoplasm through cytostomes, which transfer hemoglobin to digestive vacuoles. Hemoglobin is sequentially digested by proteases and aminopeptidases in the digestive vacuole and cytoplasm to supply *Plasmodium* with amino acids. The released heme is polymerized into hemozoin.

Iron is an essential nutrient for virtually all forms of life. Hemoglobin, being by far the most abundant reservoir of iron within humans, is thus an attractive nutrient iron source for invading pathogens. In keeping with this, numerous bacterial species have evolved systems to extract iron from host hemoglobin [3]. These systems are energetically costly and are targeted by the immune system; therefore, they are only expressed under iron-limiting conditions. In order to release hemoglobin from red blood cells, bacteria secrete toxins that lyse erythrocytes. Released hemoglobin is then bound by specific receptors that are either secreted or anchored to the cell surface of the bacteria. Upon binding of hemoglobin, these receptors remove the heme moiety from hemoglobin and pass it to heme transport proteins within the cell surface (Figure 1A and 1B). To transfer heme across the Gram-negative outer membrane, heme transport systems utilize the energy of the proton motive force. This is achieved through the TonB system, which transfers energy from the inner to the outer membrane to enable substrate transport. Once in the periplasm, heme is bound by a heme transport protein that delivers heme to the inner membrane ABC transporter, which pumps heme into the cytoplasm (Figure 1A) [4]. Gram-positive bacteria, which lack an outer membrane but contain a thick cell wall, bind and pass heme through the cell wall in a relay process with no known energy requirement (Figure 1B). Upon crossing the Gram-positive cell wall, heme is transported through the cell membrane by ABC transporters. Once in the cytoplasm of either Gram-negative or Gram-positive bacteria, heme is degraded by heme oxygenases to release iron (Figure 1A and 1B). Alternatively, intact heme can be incorporated into bacterial heme-containing proteins in a process known as molecular hijacking [5]. Bacteria are not unique in their ability to utilize hemoglobin as an iron source. Eukaryotic pathogens, including *Leishmania*, *Entamoeba*, and *Trypanosoma*, have evolved convergent mechanisms of heme-iron acquisition from this abundant host molecule [6]–[8]. Protozoa capture hemoglobin through either specific surface receptors or phagocytosis. Upon phagocytosis, the protein portion of hemoglobin is digested to release heme-iron [7]. The utilization of hemoglobin as an iron source is required for infection as demonstrated by a decrease in virulence of pathogens that are mutated for hemoglobin-iron transporters. Therefore, surface hemoglobin receptors have been studied as potential targets for vaccine development and pharmacological inhibition. Impeding a pathogen's ability to acquire iron would inhibit numerous physiological processes that are essential for viability, providing a novel avenue for antimicrobial development.

## Hemoglobin as a Source of Amino Acids for Invading Pathogens

While many microorganisms target hemoglobin to acquire heme-iron, others, such as *Plasmodium*, digest the protein as a source of amino acids. *Plasmodium* is a genus of parasitic protists and the causative agent of the disease malaria. Hemoglobin plays a central role during the blood stage of *Plasmodium* infections. Following invasion of erythrocytes, these parasites consume more than half of the cellular hemoglobin within a 24-hour period [9]. This hemoglobin consumption is achieved through several distinct mechanisms. During the initial stage of erythrocyte infection, known as the ring stage, hemoglobin is taken up by a single large vacuole in an actin-independent process known as a “big gulp” [10]. At a later trophozoit stage, hemoglobin is acquired by endocytosis of pockets of erythrocyte cytoplasm within parasite structures known as cytostomes. (Figure 1C) [9], [11]. Cytosomes then transfer hemoglobin to acidic digestive vacuoles in an actin-dependent process that is regulated by Rab5 and PfPI3K proteins [10], [12]. Late-stage parasites ingest hemoglobin through phagotrophy, which, similar to the “big gulp”, does not require actin and involves large vacuoles [10], [13]. Hemoglobin is sequentially digested by aspartic proteases, cysteine proteases, metalloproteases, and aminopeptidases in the digestive vacuole and cytoplasm of *Plasmodium* to supply the parasite with amino acids [14]. Digestion of hemoglobin has been suggested to be initiated en route to the digestive vacuole; however, the exact localization of different stages of this process is not uniformly agreed upon [15]. The critical importance of hemoglobin digestion is illustrated by the fact that blocking hemoglobin proteolysis prevents parasite development [9]. Inhibitors of hemoglobin proteases have been suggested as potential therapeutic agents against parasites that utilize hemoglobin as a source of amino acids.

## Coping with Hemoglobin Toxicity

Hemoglobin utilization leads to the release of the reactive heme co-factor from the globin portion of the protein. In *Plasmodium*, free heme is detoxified by polymerization into crystals known as hemozoin (Figure 1C). In fact, hemozoin formation is so abundant that its presence within *Anopheles gambiae* mosquitoes provided the initial clue that mosquitoes are the primary vector of malaria transmission [9]. Hemozoin formation during chronic infection manifests itself in the blackening of the spleen and liver due to the accumulation of hemozoin within these organs. Further, hemozoin appears to play a role in modulating the immune response to *Plasmodium* and is toxic to phagocytes [9]. Hemozoin crystals are generated by polymerization of heme through the formation of a bond between the iron atom of one heme molecule and carboxylate of another [16]. Dimers further polymerize through the formation of hydrogen bonds between propionates [17]. The factors that contribute to the formation of hemozoin have been the subject of considerable debate; however, lipids and proteins have been implicated in facilitating hemozoin formation within the digestive vacuoles of *Plasmodium* [18]. Antimalarial drugs such as chloroquine and possibly artemisinin inhibit hemoglobin detoxification by *Plasmodium*, underscoring the importance of this process for malarial viability [19], [20].

Bacterial pathogens utilize various strategies to reduce the toxic effects of heme. One mechanism is somewhat similar to the one utilized by *Plasmodium* whereby heme is actively sequestered by Gram-negative bacteria, thus preventing generation of reactive oxygen species [21]. Other bacterial species utilize heme oxygenases, which reduce the intracellular heme concentration through its degradation [3]. Yet other bacterial pathogens sense either heme or its toxic effects and up-regulate ATP-dependent export systems involved in heme detoxification. It is not clear whether heme itself or an unknown toxic product generated by heme is being exported; however, it is evident that both the sensing and transport components are required for heme detoxification [22].

## Human Hemoglobin Variants and Infection

Sequence variations within the hemoglobin genes profoundly influence susceptibility to infectious diseases. In this regard, hemoglobin variants have been associated with altered susceptibility to *Plasmodium*. For example, individuals who are heterozygous for the hemoglobin mutation that leads to sickle cell anemia (HbS) show increased resistance to malaria. HbS contains a glutamine to valine substitution within the beta chain of hemoglobin. In individuals that are homozygous for HbS, hemoglobin molecules aggregate within the erythrocytes, resulting in sickling of red blood cells and severe anemia. Heterozygous individuals are not anemic and eliminate up to 90% of *Plasmodium* cells within their erythrocytes. Numerous mechanisms have been suggested for decreased survival of *Plasmodium* due to HbS [23]. These include reduced growth of the parasite, increased sickling, and enhanced phagocytosis of infected erythrocytes. Recent studies attribute protection provided by HbS to a reduction in actin remodeling and cytoadherence of infected erythrocytes to capillaries, and a decrease in heme toxicity [24]–[26]. Sickle cell hemoglobin is prevalent in individuals from regions where malaria is endemic, which has created evolutionary pressure to maintain the allele within the population [27]. Other mutations resulting in hemoglobinopathies have also been found to protect against *Plasmodium* [23]. Altered susceptibility to malaria due to mutations within hemoglobin is the paradigm for how human genetics impact susceptibility to infectious diseases.

Numerous non-pathologic hemoglobin polymorphisms are found within the human population and may impact bacterial iron acquisition and virulence. This is supported by the finding that variations within the amino acid sequence of hemoglobin derived from different mammals affect iron acquisition and virulence of the bacterial pathogen *Staphylococcus aureus* [28]. A recent co-crystal structure of hemoglobin with a staphylococcal hemoglobin receptor has revealed that the region of hemoglobin recognized by this receptor is highly polymorphic within the human population. Amino acid variations in this region of hemoglobin reduce binding and utilization of hemoglobin by *S. aureus* [29]. Therefore, the susceptibility of individuals to bacterial infections may be affected by hemoglobin polymorphisms. Further, bacterial colonization may similarly be affected due to the fact that hemoglobin plays a role in this process [30]. Future identification of hemoglobin polymorphisms that influence bacterial infections may enable a personalized approach to the prevention and treatment of infectious diseases.
